# Supplementary material for: Shared Treg dysregulation underlies psoriasis vulgaris and atopic dermatitis through the PHF19-PRC2 epigenetic axis
Source: Front Immunol. 2026 Aug 20;17:1932788. doi: 10.3389/fimmu.2026.1932788 (PMC13538097; doi:10.3389/fimmu.2026.1932788)
Supplement: Supplementary file 3 [file DataSheet3.zip › Additional files/Additional file 8/02.RABEP1.leaveoneout.pdf]

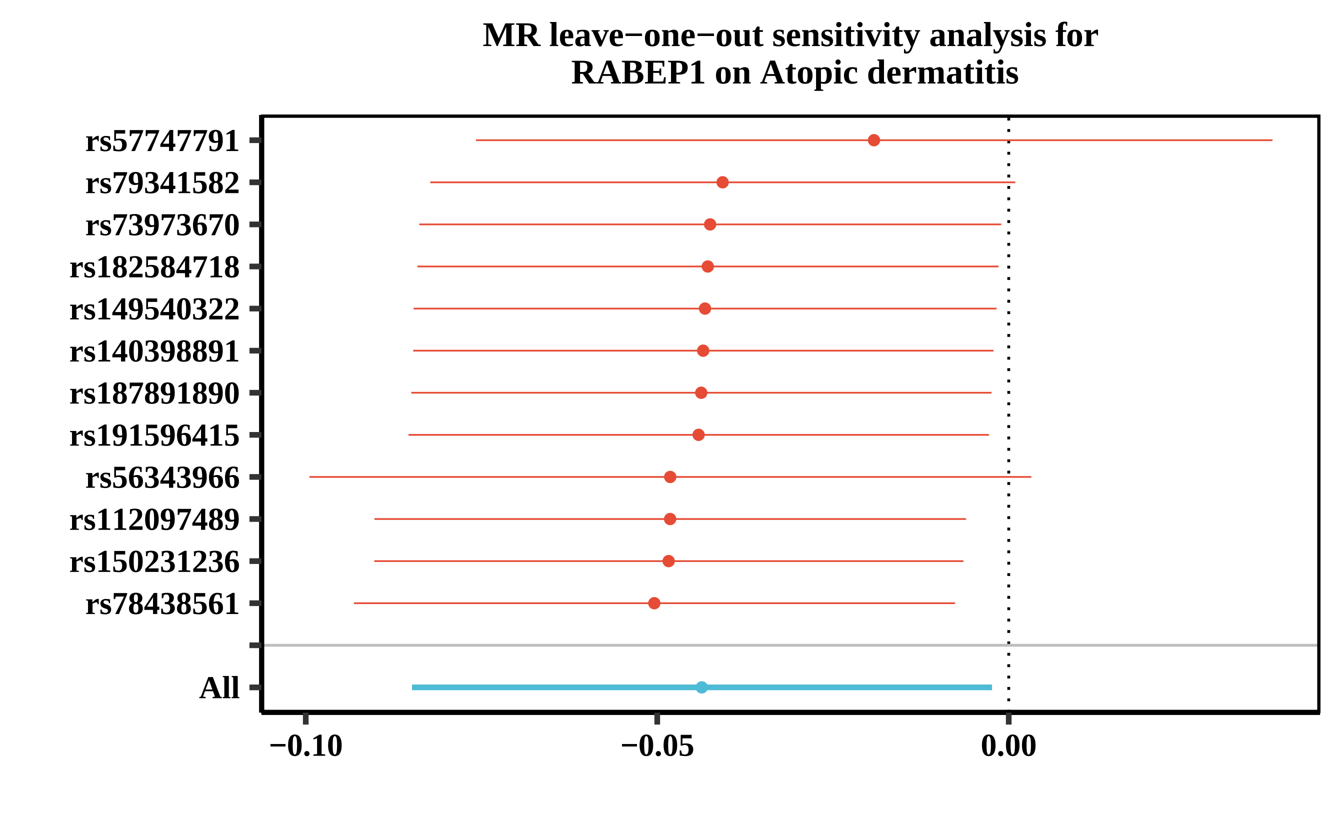
MR leave−one−out sensitivity analysis for
RABEP1 on Atopic dermatitis
rs57747791
rs79341582
rs73973670
rs182584718
rs149540322
rs140398891
rs187891890
rs191596415
rs56343966
rs112097489
rs150231236
rs78438561
All
−0.10
−0.05
0.00
